# Crystal structure of 2,3-bis­[(4-*tert*-butyl-2,6-di­methyl­phen­yl)imino]­butane

**DOI:** 10.1107/S2056989015005551

**Published:** 2015-03-25

**Authors:** Sheng-Lan Zhao, Jian-Chao Yuan, Yan Zhao

**Affiliations:** a Key Laboratory of Eco-Environment-Related Polymer Materials of the Ministry of Education, Key Laboratory of Polymer Materials of Gansu Province, College of Chemistry & Chemical Engineering, Northwest Normal University, Lanzhou 730070, People’s Republic of China

**Keywords:** crystal structure, α-di­imine ligand, catalyst, aniline, di­imino­butane

## Abstract

The title compound, C_28_H_40_N_2_, was obtained from the condensation reaction of 4-*tert*-butyl-2,6-di­methyl­aniline and butane-2,3-dione. The mol­ecule lies on an inversion centre. The C=N bond has an *E* conformation. The plane of the benzene ring is almost perpendicular to the 1,4-di­aza­butadiene mean plane [dihedral angle = 89.8 (9)°].

## Related literature   

The title compound was synthesized as an α-di­imine ligand for applications in olefin polymerization Ni^II^–α-di­imine catalysts, see: Cotts *et al.* (2000 ▸); Johnson *et al.*(1995 ▸); Ittel *et al.* (2000 ▸); Mecking *et al.* (1998 ▸) . For the effect of the ligand structure on the activity of the catalyst and the properties of the products, see: Gates *et al.* (2000 ▸); Meinhard *et al.* (2007 ▸); For related structures, see: Yuan *et al.* (2005 ▸).
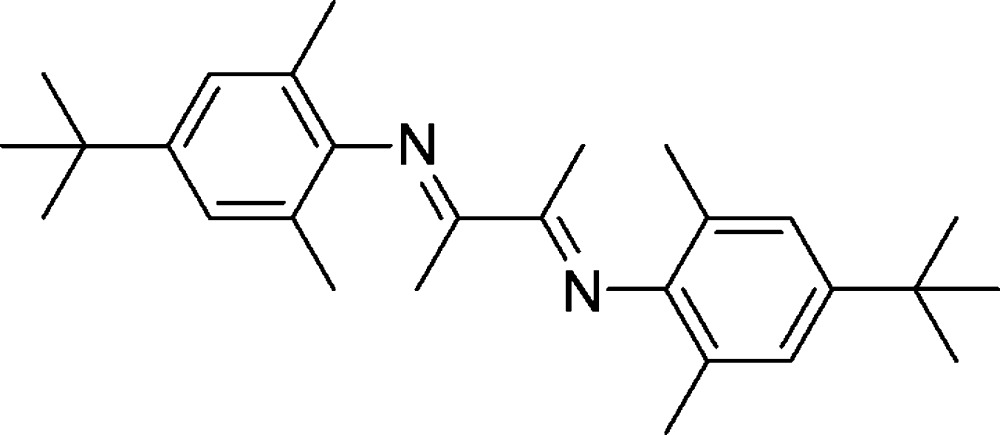


## Experimental   

### Crystal data  

C_28_H_40_N_2_
*M* *_r_* = 404.62
Triclinic, 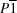

*a* = 5.993 (6) Å
*b* = 10.064 (9) Å
*c* = 11.614 (11) Å
α = 107.913 (9)°
β = 100.484 (10)°
γ = 99.260 (9)°
*V* = 637.5 (10) Å^3^
*Z* = 1
Mo *K*α radiation
μ = 0.06 mm^−1^
*T* = 296 K
0.23 × 0.21 × 0.18 mm

### Data collection  

Bruker APEXII CCD diffractometer
Absorption correction: multi-scan (*SADABS*; Bruker, 2008 ▸) *T*_min_ = 0.986, *T*_max_ = 0.989
4557 measured reflections
2310 independent reflections
1348 reflections with *I* > 2σ(*I*)
*R*_int_ = 0.030

### Refinement  

*R*[*F* ^2^ > 2σ(*F* ^2^)] = 0.102
*wR*(*F* ^2^) = 0.220
*S* = 1.05
2310 reflections
142 parameters
42 restraints
H-atom parameters constrained
Δρ_max_ = 0.52 e Å^−3^
Δρ_min_ = −0.21 e Å^−3^

### 

Data collection: *APEX2* (Bruker, 2008 ▸); cell refinement: *SAINT* (Bruker, 2008 ▸); data reduction: *SAINT*; program(s) used to solve structure: *SHELXTL* (Sheldrick, 2008 ▸); program(s) used to refine structure: *SHELXTL*; molecular graphics: *SHELXTL*; software used to prepare material for publication: *SHELXTL*.

## Supplementary Material

Crystal structure: contains datablock(s) I, New_Global_Publ_Block. DOI: 10.1107/S2056989015005551/xu5841sup1.cif

Structure factors: contains datablock(s) I. DOI: 10.1107/S2056989015005551/xu5841Isup2.hkl

Supporting information file. DOI: 10.1107/S2056989015005551/xu5841Isup3.cml

**Fig. 1.**
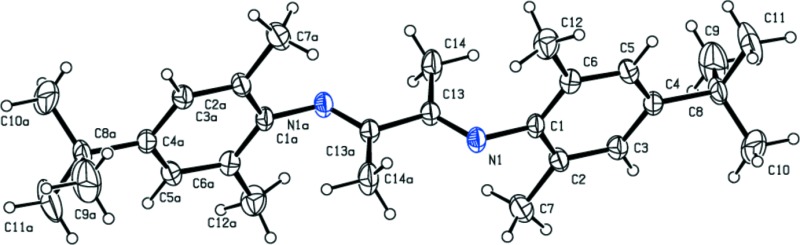
Molecular structure of the title compound, using 30% probability level ellipsoids (the hydrogens have been omitted for clarity). Primed atoms are related by the symmetry code (-x+1, -y+2, -z+1).

**Fig. 2.**
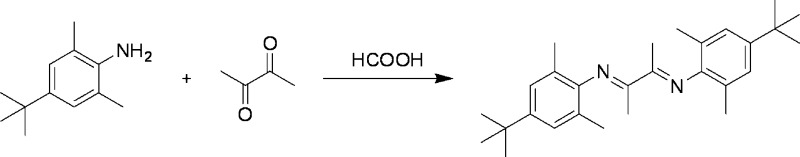
Synthesis of 2,3-bis{[4-(tert-butyl)-2,6-dimethyl]imino}butane.

. DOI: 10.1107/S2056989015005551/xu5841fig1.tif
Mol­ecular structure of the title compound, using 30% probability level ellipsoids (the hydrogens have been omitted for clarity). Primed atoms are related by the symmetry code (-x+1, −y+2, −z+1).

tert . . DOI: 10.1107/S2056989015005551/xu5841fig2.tif
Synthesis of 2,3-bis­{[4-(*tert*-but­yl)-2,6-dimeth­yl]imino}­butane*.*

CCDC reference: 1054707

Additional supporting information:  crystallographic information; 3D view; checkCIF report

## supplementary crystallographic information

### S1. Introduction

Since Brookhart and co-workers discovered Ni^II^ and Pd^II^ aryl-substituted *α-*di­imine complexes for olefin polymerization (Cotts *et al.*, 2000; Gates *et al.*, 2000; Johnson *et al.*, 1995; Meinhard *et al.*, 2007; Mecking *et al.*, 1998), late transition metal catalysts have attracted increseaing attention from their high functionality. It is well known that the ligand structure had significant influence on the product properties and polymerization activities (Ittel *et al.*, 2000; Yuan *et al.*, 2005). In this study, we designed and synthesized the title compound as a bidentate ligand, and its molecular structure was characterized by X-ray diffraction. In the crystal structure of the title compound, C_28_H_40_N_2_, the complete molecule is generated by the application of *C*_2_ symmetry. The single bond of 1,4-di­aza­butadiene fragment is (E)-configured. The dihedral angle between the benzene ring and 1,4-di­aza­butadiene plane is 89.8 (9)°.

### S2. Synthesis and crystallization

Formic acid (0.2 ml) was added to a stirred solution of butane-2,3-dione (0.09 g, 1.00 mmol) and 4-(*tert*-butyl)-2,6-di­methyl­aniline (0.39 g, 2.2 mmol) in ethanol (10 ml). The mixture was refluxed for 24 h, then cooled and the precipitate was separated by filtration. The solid was recrystallized from MeOH/CH_2_Cl_2_ (v/v = 10:1), than washed with cold ethanol and dried under vacuum (0.35 g, 87% yield). Anal. Calc. for C_28_H_40_N_2_: C, 83.11; H, 9.96; N, 6.92. Found: C, 83.23; H, 10.03; N, 6.89.

### S3. Refinement

All hydrogen atoms were placed in calculated positions with C—H distances of 0.93Å and 0.96Å for aryl and methyl H atoms. They were included in the refinement in a riding model approximation, respectively. The H atoms were assigned *U*_iso_ = 1.2U_eq_(C) for aryl H and *U*_iso_ = 1.5U_eq_(C) for methyl H.

### Crystal data

**Table:**

C_28_H_40_N_2_	*Z* = 1
*M**_r_* = 404.62	*F*(000) = 222
Triclinic, *P*1	*D*_x_ = 1.054 Mg m^−^^3^
Hall symbol: -P 1	Mo *K*α radiation, λ = 0.71073 Å
*a* = 5.993 (6) Å	Cell parameters from 1229 reflections
*b* = 10.064 (9) Å	θ = 2.4–28.3°
*c* = 11.614 (11) Å	µ = 0.06 mm^−^^1^
α = 107.913 (9)°	*T* = 296 K
β = 100.484 (10)°	Block, yellow
γ = 99.260 (9)°	0.23 × 0.21 × 0.18 mm
*V* = 637.5 (10) Å^3^	

### Data collection

**Table:**

Bruker APEXII CCD diffractometer	2310 independent reflections
Radiation source: fine-focus sealed tube	1348 reflections with *I* > 2σ(*I*)
Graphite monochromator	*R*_int_ = 0.030
φ and ω scans	θ_max_ = 25.5°, θ_min_ = 2.4°
Absorption correction: multi-scan (*SADABS*; Bruker, 2008)	*h* = −7→7
*T*_min_ = 0.986, *T*_max_ = 0.989	*k* = −12→11
4557 measured reflections	*l* = −14→13

### Refinement

**Table:**

Refinement on *F*^2^	Primary atom site location: structure-invariant direct methods
Least-squares matrix: full	Secondary atom site location: difference Fourier map
*R*[*F*^2^ > 2σ(*F*^2^)] = 0.102	Hydrogen site location: inferred from neighbouring sites
*wR*(*F*^2^) = 0.220	H-atom parameters constrained
*S* = 1.05	*w* = 1/[σ^2^(*F*_o_^2^) + (0.040*P*)^2^ + 1.2419*P*] where *P* = (*F*_o_^2^ + 2*F*_c_^2^)/3
2310 reflections	(Δ/σ)_max_ < 0.001
142 parameters	Δρ_max_ = 0.52 e Å^−^^3^
42 restraints	Δρ_min_ = −0.21 e Å^−^^3^

### Special details

**Table:**

Geometry. All e.s.d.'s (except the e.s.d. in the dihedral angle between two l.s. planes) are estimated using the full covariance matrix. The cell e.s.d.'s are taken into account individually in the estimation of e.s.d.'s in distances, angles and torsion angles; correlations between e.s.d.'s in cell parameters are only used when they are defined by crystal symmetry. An approximate (isotropic) treatment of cell e.s.d.'s is used for estimating e.s.d.'s involving l.s. planes.
Refinement. Refinement of *F*^2^ against ALL reflections. The weighted *R*-factor *wR* and goodness of fit *S* are based on *F*^2^, conventional *R*-factors *R* are based on *F*, with *F* set to zero for negative *F*^2^. The threshold expression of *F*^2^ > σ(*F*^2^) is used only for calculating *R*-factors(gt) *etc*. and is not relevant to the choice of reflections for refinement. *R*-factors based on *F*^2^ are statistically about twice as large as those based on *F*, and *R*- factors based on ALL data will be even larger.

### Fractional atomic coordinates and isotropic or equivalent isotropic displacement parameters (Å^2^)

**Table:**

	*x*	*y*	*z*	*U*_iso_*/*U*_eq_	
C1	0.4381 (7)	0.6834 (4)	0.3770 (4)	0.0394 (10)	
C2	0.2730 (7)	0.6166 (4)	0.2638 (4)	0.0434 (11)	
C3	0.2889 (7)	0.4854 (4)	0.1863 (4)	0.0439 (11)	
H3	0.1786	0.4407	0.1104	0.053*	
C4	0.4620 (7)	0.4180 (4)	0.2170 (4)	0.0413 (11)	
C5	0.6199 (8)	0.4869 (4)	0.3302 (4)	0.0466 (11)	
H5	0.7373	0.4430	0.3531	0.056*	
C6	0.6132 (8)	0.6185 (4)	0.4121 (4)	0.0431 (11)	
C7	0.0812 (9)	0.6857 (5)	0.2255 (5)	0.0642 (15)	
H7A	−0.0024	0.7076	0.2894	0.096*	
H7B	−0.0239	0.6209	0.1487	0.096*	
H7C	0.1475	0.7725	0.2138	0.096*	
C8	0.4746 (9)	0.2729 (4)	0.1263 (4)	0.0540 (13)	
C9	0.5397 (13)	0.2937 (6)	0.0139 (6)	0.107 (2)	
H9A	0.5464	0.2030	−0.0431	0.160*	
H9B	0.6895	0.3591	0.0387	0.160*	
H9C	0.4250	0.3322	−0.0264	0.160*	
C10	0.2528 (12)	0.1679 (6)	0.0939 (8)	0.138 (3)	
H10A	0.1292	0.2030	0.0567	0.208*	
H10B	0.2225	0.1533	0.1680	0.208*	
H10C	0.2614	0.0785	0.0357	0.208*	
C11	0.6673 (13)	0.2145 (6)	0.1849 (6)	0.112 (2)	
H11A	0.6383	0.2030	0.2603	0.167*	
H11B	0.8149	0.2805	0.2039	0.167*	
H11C	0.6703	0.1233	0.1274	0.167*	
C12	0.7936 (9)	0.6874 (5)	0.5336 (4)	0.0648 (15)	
H12A	0.9289	0.7412	0.5211	0.097*	
H12B	0.8363	0.6146	0.5645	0.097*	
H12C	0.7313	0.7507	0.5930	0.097*	
C13	0.5168 (8)	0.9330 (4)	0.4552 (4)	0.0404 (10)	
C14	0.6647 (10)	0.9478 (5)	0.3680 (5)	0.0671 (16)	
H14A	0.6877	0.8553	0.3238	0.101*	
H14B	0.8131	1.0115	0.4145	0.101*	
H14C	0.5890	0.9860	0.3095	0.101*	
N1	0.4167 (6)	0.8151 (3)	0.4603 (3)	0.0440 (10)	

### Atomic displacement parameters (Å^2^)

**Table:**

	*U*^11^	*U*^22^	*U*^33^	*U*^12^	*U*^13^	*U*^23^
C1	0.052 (3)	0.026 (2)	0.042 (2)	0.0086 (19)	0.023 (2)	0.0084 (18)
C2	0.050 (3)	0.034 (2)	0.051 (3)	0.016 (2)	0.020 (2)	0.013 (2)
C3	0.047 (3)	0.037 (2)	0.044 (3)	0.010 (2)	0.010 (2)	0.009 (2)
C4	0.049 (3)	0.031 (2)	0.047 (3)	0.010 (2)	0.019 (2)	0.0123 (19)
C5	0.050 (3)	0.037 (2)	0.054 (3)	0.018 (2)	0.013 (2)	0.013 (2)
C6	0.051 (3)	0.036 (2)	0.043 (3)	0.009 (2)	0.013 (2)	0.013 (2)
C7	0.063 (3)	0.053 (3)	0.072 (4)	0.026 (3)	0.012 (3)	0.013 (3)
C8	0.068 (3)	0.033 (2)	0.061 (3)	0.021 (2)	0.029 (2)	0.005 (2)
C9	0.164 (6)	0.077 (4)	0.080 (4)	0.040 (4)	0.058 (4)	0.008 (3)
C10	0.107 (5)	0.049 (3)	0.198 (7)	−0.008 (3)	0.068 (5)	−0.046 (4)
C11	0.148 (6)	0.067 (4)	0.108 (5)	0.068 (4)	0.018 (4)	0.000 (3)
C12	0.072 (4)	0.054 (3)	0.052 (3)	0.012 (3)	−0.002 (3)	0.007 (2)
C13	0.050 (3)	0.032 (2)	0.042 (2)	0.0111 (19)	0.018 (2)	0.0119 (18)
C14	0.102 (4)	0.035 (2)	0.074 (3)	0.017 (3)	0.053 (3)	0.013 (2)
N1	0.057 (2)	0.0297 (19)	0.047 (2)	0.0110 (17)	0.0237 (18)	0.0097 (16)

### Geometric parameters (Å, º)

**Table:**

C1—C2	1.388 (6)	C9—H9A	0.9600
C1—C6	1.386 (6)	C9—H9B	0.9600
C1—N1	1.421 (5)	C9—H9C	0.9600
C2—C3	1.379 (5)	C10—H10A	0.9600
C2—C7	1.505 (6)	C10—H10B	0.9600
C3—C4	1.380 (6)	C10—H10C	0.9600
C3—H3	0.9300	C11—H11A	0.9600
C4—C5	1.372 (6)	C11—H11B	0.9600
C4—C8	1.538 (5)	C11—H11C	0.9600
C5—C6	1.384 (6)	C12—H12A	0.9600
C5—H5	0.9300	C12—H12B	0.9600
C6—C12	1.497 (6)	C12—H12C	0.9600
C7—H7A	0.9600	C13—N1	1.264 (5)
C7—H7B	0.9600	C13—C14	1.486 (6)
C7—H7C	0.9600	C13—C13^i^	1.497 (7)
C8—C10	1.465 (7)	C14—H14A	0.9600
C8—C9	1.493 (7)	C14—H14B	0.9600
C8—C11	1.523 (7)	C14—H14C	0.9600
			
C2—C1—C6	120.6 (3)	H9A—C9—H9B	109.5
C2—C1—N1	119.1 (4)	C8—C9—H9C	109.5
C6—C1—N1	120.2 (4)	H9A—C9—H9C	109.5
C1—C2—C3	118.6 (4)	H9B—C9—H9C	109.5
C1—C2—C7	121.0 (4)	C8—C10—H10A	109.5
C3—C2—C7	120.4 (4)	C8—C10—H10B	109.5
C4—C3—C2	122.6 (4)	H10A—C10—H10B	109.5
C4—C3—H3	118.7	C8—C10—H10C	109.5
C2—C3—H3	118.7	H10A—C10—H10C	109.5
C5—C4—C3	116.9 (4)	H10B—C10—H10C	109.5
C5—C4—C8	122.5 (4)	C8—C11—H11A	109.5
C3—C4—C8	120.6 (4)	C8—C11—H11B	109.5
C4—C5—C6	123.2 (4)	H11A—C11—H11B	109.5
C4—C5—H5	118.4	C8—C11—H11C	109.5
C6—C5—H5	118.4	H11A—C11—H11C	109.5
C1—C6—C5	118.0 (4)	H11B—C11—H11C	109.5
C1—C6—C12	122.0 (4)	C6—C12—H12A	109.5
C5—C6—C12	120.0 (4)	C6—C12—H12B	109.5
C2—C7—H7A	109.5	H12A—C12—H12B	109.5
C2—C7—H7B	109.5	C6—C12—H12C	109.5
H7A—C7—H7B	109.5	H12A—C12—H12C	109.5
C2—C7—H7C	109.5	H12B—C12—H12C	109.5
H7A—C7—H7C	109.5	N1—C13—C14	124.9 (4)
H7B—C7—H7C	109.5	N1—C13—C13^i^	116.8 (5)
C10—C8—C9	112.2 (6)	C14—C13—C13^i^	118.2 (5)
C10—C8—C11	108.4 (5)	C13—C14—H14A	109.5
C9—C8—C11	105.7 (5)	C13—C14—H14B	109.5
C10—C8—C4	110.2 (4)	H14A—C14—H14B	109.5
C9—C8—C4	109.4 (4)	C13—C14—H14C	109.5
C11—C8—C4	110.9 (4)	H14A—C14—H14C	109.5
C8—C9—H9A	109.5	H14B—C14—H14C	109.5
C8—C9—H9B	109.5	C13—N1—C1	120.1 (3)
			
C6—C1—C2—C3	−0.9 (6)	N1—C1—C6—C12	−3.9 (6)
N1—C1—C2—C3	−177.0 (4)	C4—C5—C6—C1	−0.2 (6)
C6—C1—C2—C7	179.5 (4)	C4—C5—C6—C12	−179.4 (4)
N1—C1—C2—C7	3.5 (6)	C5—C4—C8—C10	125.0 (6)
C1—C2—C3—C4	0.1 (6)	C3—C4—C8—C10	−55.4 (7)
C7—C2—C3—C4	179.7 (4)	C5—C4—C8—C9	−111.2 (5)
C2—C3—C4—C5	0.6 (6)	C3—C4—C8—C9	68.3 (6)
C2—C3—C4—C8	−179.0 (4)	C5—C4—C8—C11	5.0 (6)
C3—C4—C5—C6	−0.5 (6)	C3—C4—C8—C11	−175.5 (5)
C8—C4—C5—C6	179.0 (4)	C14—C13—N1—C1	−1.2 (7)
C2—C1—C6—C5	0.9 (6)	C13^i^—C13—N1—C1	179.2 (5)
N1—C1—C6—C5	176.9 (4)	C2—C1—N1—C13	−91.4 (5)
C2—C1—C6—C12	−179.9 (4)	C6—C1—N1—C13	92.6 (5)

Symmetry code: (i) −*x*+1, −*y*+2, −*z*+1.
