# Supplementary figures and images for: Crystal structure of 2,3-bis­[(4-tert-butyl-2,6-di­methyl­phen­yl)imino]­butane
Source: Acta Crystallogr E Crystallogr Commun. 2015 Mar 25;71(Pt 4):o258. doi: 10.1107/S2056989015005551 (PMC4438850; doi:10.1107/S2056989015005551)

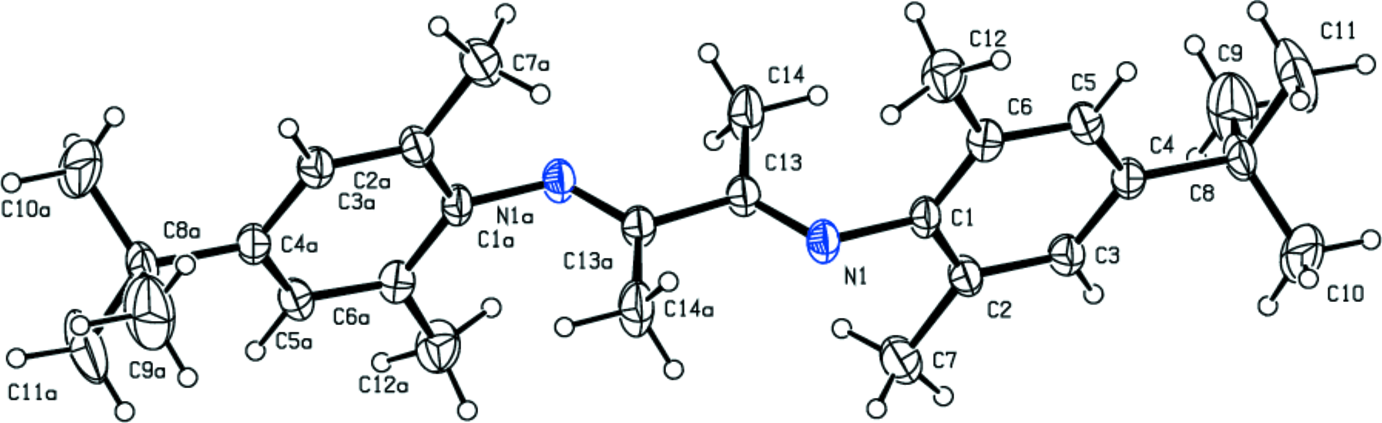

Supplement: Supplementary file 4 [file e-71-0o258-fig1.tif]
